# Soluble Dietary Fiber, One of the Most Important Nutrients for the Gut Microbiota

**DOI:** 10.3390/molecules26226802

**Published:** 2021-11-11

**Authors:** Zhi-Wei Guan, En-Ze Yu, Qiang Feng

**Affiliations:** 1 Shandong Provincial Key Laboratory of Oral Tissue Regeneration, Shandong Engineering Laboratory for Dental Materials and Oral Tissue Regeneration, Department of Human Microbiome, School of Stomatology, Shandong University, Jinan 250012, China; guanzhiwei1109@163.com (Z.-W.G.); yuenze31415@163.com (E.-Z.Y.); 2 School of Life Science, Qi Lu Normal University, Jinan 250200, China; 3 State Key Laboratory of Microbial Technology, Shandong University, Qingdao 266237, China

**Keywords:** soluble dietary fiber, resistant oligosaccharide, viscous fiber, gut microbiota, human health

## Abstract

Dietary fiber is a widely recognized nutrient for human health. Previous studies proved that dietary fiber has significant implications for gastrointestinal health by regulating the gut microbiota. Moreover, mechanistic research showed that the physiological functions of different dietary fibers depend to a great extent on their physicochemical characteristics, one of which is solubility. Compared with insoluble dietary fiber, soluble dietary fiber can be easily accessed and metabolized by fiber-degrading microorganisms in the intestine and produce a series of beneficial and functional metabolites. In this review, we outlined the structures, characteristics, and physiological functions of soluble dietary fibers as important nutrients. We particularly focused on the effects of soluble dietary fiber on human health via regulating the gut microbiota and reviewed their effects on dietary and clinical interventions.

## 1. Introduction

The dietary pattern is closely related to human health. Hu et al. identified two major dietary patterns, which are the Prudent and Western diets [1]. The Prudent diet is considered healthy and is characterized by higher intake of vegetables, fruit, legumes, whole grains, fish, and poultry. Compared to the Prudent diet, the Western diet is characterized by abundant red meat, fat, and refined carbohydrates [2,3]. The Western diet contributes to increased risk of non-communicable diseases, especially gastrointestinal diseases and metabolic diseases which are partially attributed to the deficiency of dietary fiber in the Western diet [4,5,6,7,8,9,10]. According to the widely accepted definition derived from Codex Alimentarius Alinorm in 2009, dietary fiber is considered as edible carbohydrate polymers with three or more monomeric units that are resistant to endogenous digestive enzymes and thus are neither hydrolyzed nor absorbed in the small intestine [11]. Based on their structures, dietary fibers can be classified into non-starch polysaccharides, resistant starches (RSs), and resistant oligosaccharides. Moreover, the non-carbohydrate polymer, lignin, which coexists with cellulose in plant cell walls, is also considered in this definition as dietary fiber. Based on the source, dietary fibers are mainly comprised three subgroups: (i) Carbohydrate polymers naturally existing in edible plants and consumed as vegetables, fruits, seeds, cereals, and tubers; (ii) edible carbohydrate polymers obtained from raw foods by physical, enzymatic, and chemical means that have a proven physiological benefit (e.g., resistant oligosaccharides, inulin, and psyllium); and (iii) synthetic carbohydrate polymers with a proven physiological benefit (e.g., methylcellulose). [11,12].

**Figure 1:**
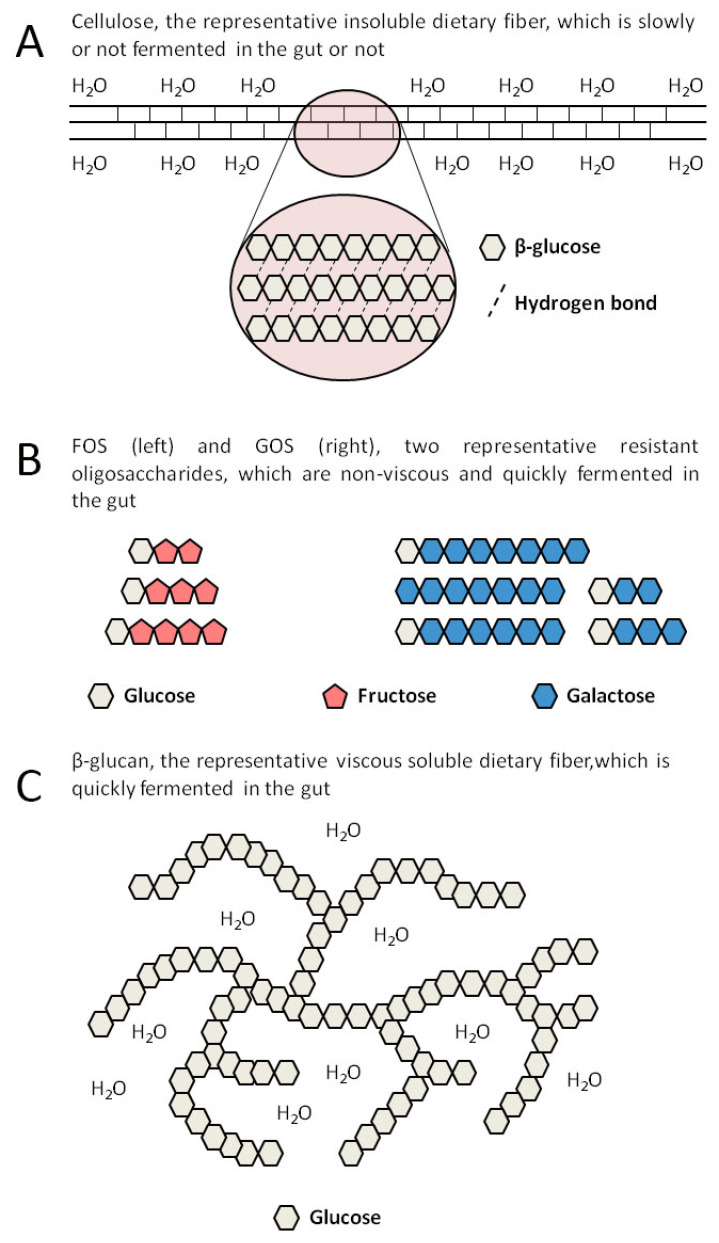
Schematic diagram of the structures of representative insoluble dietary fiber and SDF. (**A**). The representative insoluble dietary fiber, cellulose. (**B**). The representative resistant oligosaccharides, FOS and GOS. (**C**). The representative viscous SDF, β-glucan.

Dietary fibers from food pass through the small intestine into the large intestine, where they play physiological roles. Dietary fibers contain a variety of organic polymers, with different monomers linked by different glycosidic bonds, showing complex and heterogeneous structure [13]. To help correlate physicochemical characteristics of dietary fiber with their physiological functions, many ways in classifying dietary fiber were established, which include solubility, viscosity, and fermentability [14]. Depending on solubility, dietary fiber can be categorized as insoluble or soluble (SDFs) [15]. The sugar chains in insoluble dietary fiber associate with each other by dense hydrogen bonds, forming a hydrophobic and crystalline structure, which can resist the hydrolysis of exogenous glucosidases. As the most widely distributed and abundant insoluble fiber in nature, cellulose is a polysaccharide with high molecular weight, composed of β-glucose. It is the main structural component of plant cell walls, which usually combines with hemicellulose, pectin, and lignin [16]. A schematic diagram of molecular structure of cellulose is shown in Figure 1A. Most insoluble dietary fibers, such as cellulose, hemi-cellulose, and lignin, have an effect on bulking fecal material, but are not or just slowly utilized by gut bacteria. On the contrary, SDFs can be readily and quickly metabolized by gut bacteria, in the process of which SDFs significantly influence the abundance and diversity of the human gut microbiota [17]. Studies confirmed that dietary fibers, especially SDFs, can positively regulate the gut microbiota and be metabolized to beneficial products, mainly short-chain fatty acids (SCFAs), thus providing many advantages to human health, such as reducing the risk of gastrointestinal diseases including irritable bowel syndrome (IBS), inflammatory bowel disease (IBD), diverticular disease, functional constipation, fecal incontinence, and colorectal cancer (CRC) [18,19,20]. 

Here, we summarized the structures and characteristics of SDFs as well as their effects on the gut microbiota, by which they help to improve human health. We hope that this review can help the readers learn about SDFs and their influence on the gut microbiota, and provide some advice for future research in related fields.

## 2. SDFs Include Many Different Substances

Solubility means the ability of dietary fibers to dissolve in water. Compared to insoluble dietary fibers, SDFs have a high affinity for water. SDFs includes various active substances with different structures, which are mainly composed of resistant oligosaccharides and viscous dietary fibers with a high molecular weight [21]. It is worth noting that solubility can be quite variable depending on not only the structures of fibers but also the external factors such as temperature and pH value [13]. For example, the solubility of pectin improves with the increase of the abundance of its side chains [22]. In addition, some viscous fibers are obtained via chemically modifying insoluble fibers, such as methylcellulose and type IV RS (RS-4), which were produced from cellulose and type II RS, respectively [23,24]. Significantly, SDFs include a large amount of active substances showing the structural complexity, which can be administered in the form of natural foods or dietary supplements [25]. In consideration of the fact that certain dietary fibers, including most SDFs, have the effect of promoting the proliferation of specific probiotics, they are also called microbiota-accessible carbohydrates (MACs) or prebiotics [26]. The definition of prebiotic published in 2017 is a substrate that is selectively utilized by host microorganisms conferring a health benefit [27]. The structures, sources, and physicochemical characteristics of common SDFs are shown in Table 1.

Resistant oligosaccharides, which are also called functional oligosaccharides, refer to oligosaccharides with a degree of polymerization (DP) from 3 to 9 that have prebiotic effects. Fructo-oligosaccharides (FOS) and galacto-oligosaccharides (GOS) are the most studied and typical resistant oligosaccharides. They are easily fermented in the gut because of their low molecular weight and high solubility [28]. A schematic diagram of molecular structures of FOS and GOS is shown in Figure 1B. The preparation of resistant oligosaccharides can be achieved by enzymatic methods, such as hydrolysis and isomerization, and chemical methods [29,30].

Inulin is a chain polysaccharide composed of D-fructose linked by a β-glycosidic bond with a glucose at the end, and with a DP from 2 to 60 [31]. Inulins with a DP less than 10 belong to resistant oligosaccharides, while the others belong to viscous dietary fibers. Due to their large molecular weight and strong hydrophilicity, viscous fibers, including β-glucan, pectins, and gums, can dissolve in water as well as forming a gelatinous structure at the required critical concentration with a water-holding capacity, which can inhibit the absorption of glucose and lipids in the gut [17]. β-glucan is composed of D-glucose linked by a β-glycosidic bond, which is the main structural component of plant cell walls and can also be synthesized by enzyme technology [32]. A schematic diagram of the structure of viscous β-glucan is shown in Figure 1C. Pectin, a hetero-polysaccharide widely existing in the middle lamella and primary cell walls of the plants, is mainly composed of D-galacturonic acids linked by an α-1,4-glycosidic bond and contains a small amount of rhamnose, arabinose, and galactose [33]. Gums are high-molecular-weight carbohydrates that at low concentrations can combine with water to form gels. There are many types of gums, such as exudate gums (e.g., acacia gum), mucilage gums (e.g., psyllium), and microbial gums (e.g., xanthan gum). Exudate gums are the exudates of certain trees and shrubs, which are wildly applied in the food and other industries because they are easy to produce [34].

Most viscous dietary fibers are rapidly and completely fermented in water with the gelatinous structure disappearing, but there are exceptions, such as psyllium. Psyllium, a widely concerned and studied dietary gum, is a powder ground from the seeds of Plantago ovate. Psyllium is rich in mucilage, which is a mixture of polysaccharides consisting of pentoses, hexoses, and uronic acids [35]. The results of in vitro experiments showed that psyllium is a kind of fermentable fiber, but in vivo experiments confirmed that psyllium can hardly be fermented [36]. Therefore, compared with other fermentable viscous fibers, psyllium can always retain its water-holding capacity in the gut, which is the reason why psyllium is used as a laxative in clinics [35]. 

Methylcellulose is the simplest cellulose derivative. It is made by alkalizing cellulose and then methylating alkaline cellulose by chloromethane, in the process of which methyl groups substitute the hydrogens of the hydroxyls at C-2, C-3, and/or C-6 positions of β-D-glucose units in cellulose. Finally, the product is purified and ground to a powder. Methylcellulose is approved as a thickener and gelling additive in food processing in many countries [37]. As a viscous dietary fiber, methylcellulose is completely non-fermentable in the gut, but has the function of relaxing the bowel [38]. 

RS is a kind of starch, which can escape digestion in the small intestine and then arrives in the colon where it can be utilized by certain specialized bacteria [39]. RS is divided into five subtypes. Although all types of RSs exhibit prebiotic activity, only RS-4 is soluble. RS-4 is a group of starches that are chemically modified by conversion, substitution, or cross-linking in order to lower their digestibility in the small intestine [40].

**Table 1:** The structures, sources, and physicochemical characteristics of common SDFs and their effects on the gut microbiota.

Type	Structure	Source	Viscosity	Fermentability	Changes Related to the Gut Microbiota
FOS	Sucrose combines with 1 to 3 fructoses linked by a β-glycosidic bond	Vegetables, fruits, produced by enzyme-catalyzed synthesis	No	Yes	Increased α-diversity, Increased *Bifidobacteria* and *Lactobacilli* [41]
GOS	Galactose or glucose combines with 1 to 7 galactoses linked by a β-glycosidic bond	Milk, produced by enzyme-catalyzed synthesis	No	Yes	Increased β-diversity, Increased Lactobacillaceae and Lachnospiraceae, Decreased Ruminococcaceae [42]
Inulin	D-fructose linked by a β-glycosidic bond with glucose at the end	Vegetables, fruits, grains	DP < 9: No DP ≥ 9: Yes	Yes	Increased β-diversity, Increased Prevotellaceae [43] Increased α-diversity, Increased *Bifidobacteria*, Decreased *Desulfovibrio* [44]
β-glucan	High polymer composed of D-glucose linked by a β-glycosidic bond	Grains	Yes	Yes	Increased *Bifidobacteria* and *Lactobacilli*, Decreased *Enterobacteriaceae* [45]
Pectins	Polysaccharides with complex structures containing D-galacturonic acid, rhamnose, arabinose, and galactose	Vegetables, fruits, beans	Yes	Yes	Increased *Bifidobacteria*, *Lactobacilli*, and *Faecalibaculum* spp. [46] Increased β-diversity, Inhibited *Citrobacter rodentium* [47]
Gums	Polysaccharides with complex structures containing mannose, galactose, glucose, and D-galacturonic acid	Leguminous plants, nuts, seaweeds	Yes	Yes	Increased *Bifidobacteria* and *Lactobacilli* [48] Inhibited *Clostridium histolyticum* [49]
Psyllium	Mixture of polysaccharides consisting of arabinose, xylose, galactose, rhamnose, and D-galacturonic acid	*Plantago ovate*	Yes	No	No
Methylcellulose	Long-chain substituted cellulose, in which about 30% of the hydroxyl groups exist in the form of methoxyl	Synthesized	Yes	No	No
RS-4	Chemically modified starch, such as acetyl starch, hydroxypropyl starch, heat-modified starch, and phosphorylated starch	Synthesized	Yes	Yes	Increased *Bacteroides*, *Bifidobacteria*, *Lactobacilli*, *Coprococcus*, and *Allobaculum* [50,51,52]

FOS: fructo-oligosaccharides, GOS: galacto-oligosaccharides, DP: degree of polymerization, RS-4: type IV resistant starch.

## 3. Mechanism in the Utilization of SDFs by the Gut Microbiota

The human body is occupied by trillions of microorganisms, most of which are bacteria [53]. They are closely related to human health and the gut is their most densely populated habitat [54]. In 2020, Almeida et al. established the Unified Human Gastrointestinal Genome (UHGG) collection by editing and analyzing previous human gut microbiome datasets. UHGG consists of 204,938 non-redundant genomes from 4644 gut prokaryotes and is the most comprehensive sequence database of the human gut microbiome so far [55]. Firmicutes and Bacteroidetes are the dominant phyla in the gut microbiota [56]. The digestive enzymes of the human body cannot degrade SDFs. When SDFs in foods enter the colon though the small intestine, the gut bacteria can degrade SDFs into oligosaccharides or monosaccharides through different degradation systems, and then absorb them by specific transport systems for energy source [57]. Although each organism contains relatively few cellulolytic enzymes, the intestinal microbes in total contain about 130 glycoside hydrolase (GH) families, 22 polysaccharides lyase (PL) families, and 16 carbohydrate esterase families, which provide the microbiota with the flexibility to switch between different fiber energy sources [58].

**Figure 2:**
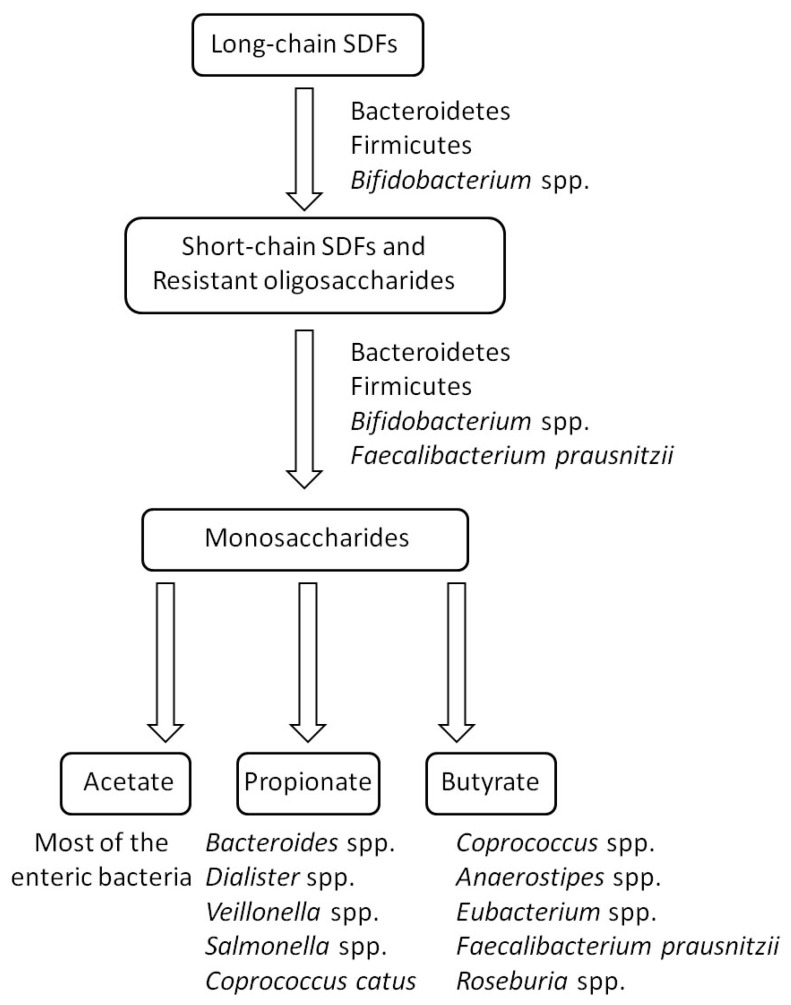
Schematic diagram of the process of degrading and metabolizing SDFs by the gut microbiota.

In terms of the degradation of long-chain dietary fibers, in vitro studies by co-culturing with human fecal samples indicated that the species from the phylum Firmicutes (e.g., *Ruminococcus bromii*, *Eubacterium rectale*, *Clostridium* spp., and *Roseburia* spp.), *Bifidobacterium* spp., and *Bacteroides* spp., are the major degraders of RSs [59,60]. However, bioinformatic analysis of a model human microbiome constructed from 177 human microbial genomes revealed that the bacteria from the phylum Bacteroidetes are the likely primary degraders of the various complex polysaccharides in the plant cell walls [61]. Many gut microbes evolved diverse strategies to utilize SDFs. For example, *Bacteroides*, the typical Gram-negative bacteria, is the most widely studied microbe in the field of polysaccharide transport and utilization of the gut microbiota due to its efficient polysaccharide degradation system, which may be the reason that *Bacteroides* is the dominant bacteria in the gut microbiota. About 20% of the genes in the genome of *Bacteroides* are involved in the utilization of polysaccharides [57]. Its transportation and decomposition of polysaccharides require the participation of multiple functional proteins and the system containing these proteins is called the starch utilization system (Sus). Sus consists of eight components, SusA-SusG and SusR, which are responsible for the detection, binding, hydrolysis, and transport of exogenous polysaccharides, respectively [62]. Notably, all *Bacteroides* possess the orthologous components of the Sus system, many of which have been demonstrated to be responsible for the absorption and utilization of specific SDFs such as inulin and pectin [63]. Moreover, certain Gram-positive bacteria in the gut microbiota are also prominent in the process of glycan utilization. The most representative Gram-positive intestinal saccharolytic microbe is the *Bifidobacterium* genus from the Actinobacteria phylum [64]. Bioinformatics analyses showed that the genes encoding modules involved in carbohydrate utilization account for nearly 13% of the identified genes in the bifidobacterial genome [65]. Unlike Gram-negative bacteria, Gram-positive *Bifidobacteria* have no periplasmic space. Therefore, complex polysaccharides first need to be digested into oligosaccharides extracellularly by the GHs anchored on the cell surface. Then, the produced oligosaccharides are transported into the cytoplasm for further degradation or shared by other members in the gut microbiota as nutritional sources [66]. The bacterial ability to degrade fibers is relative to the chain length of fibers. Resistant oligosaccharides are usually easier to degrade than polysaccharides. Many bacteria in the gut microbiota can utilize short-chain fibers. Besides the degraders of long-chain fibers, *Faecalibacterium prausnitzii* and *Lactobacillus* spp. were found to be able to utilize FOS in vitro [67]. Except for the decomposition of SDFs, the subsequent production of SCFAs is also necessary for human health. SCFAs are the fatty acids with 1 to 6 carbon atoms, mainly including acetate, propionate, and butyrate. Succinate and lactate are also produced by intestinal microbes, but they are usually regarded as intermediates due to their small amounts and the conversion of them to SCFAs by other microorganisms. The current findings have demonstrated that the health benefits of SDFs depend more on SCFAs [15,20]. Many intestinal bacteria have been proved to be specific SCFA producers, although some of them have a poor ability to degrade SDFs (e.g., *Anaerostipes* spp., *Coprococcus* spp., *Dialister* spp., *Veillonella* spp., and *Salmonella* spp.) [20,68]. The process of degrading and metabolizing SDFs by the gut microbiota is illustrated as Figure 2.

The above data are indicative of the link between the ability of the intestinal bacteria in glycan utilization and its corresponding ecological niche in the human gut. It also makes SDF metabolism closely related to human health.

## 4. Effects of SDFs on the Gut Microbiota

Dietary fibers, especially SDFs, provide the main carbon and energy source for the gut microbiota. SDFs have prebiotic effects by increasing the beneficial bacteria and improving the intestinal environment [69].

For example, FOS can effectively increase the bacterial diversity of the human gut microbiota and improve the abundance of *Bifidobacteria* and *Lactobacilli* [41]. The results of the study by Yang et al. showed that the administration of GOS is conductive to improving neuro-inflammatory and cognitive dysfunction in a rat model of abdominal surgery. Furthermore, in-depth fecal microbiota analysis by 16S rRNA sequencing revealed that the administration of GOS can induce a significant increase in β-diversity of the gut microbiota and the proliferation of *Bifidobacterium* and other potentially anti-inflammatory bacteria [42]. Inulin was confirmed to be beneficial to health by regulating the gut microbiota. In *ob*/*ob* mice, it was found that an inulin-containing diet can improve fat accumulation and glucose intolerance. Further fecal microbiota analysis displayed that the β-diversity of the *ob*/*ob* mice tended to the level of the wild type mice and the Prevotellaceae UCG 001 family was significantly enriched, which positively affected the leptin-related pathways in the *ob*/*ob* mice [43]. In a randomized controlled trial on healthy adults, the addition of agave inulin bettered the diversity and activity of the gut microbiota including increasing *Bifidobacteria* and depleting *Desulfovibrio* [44]. Cereal is the main dietary source of β-glucan. A study in rats showed that the administration of cereal β-glucan can promote the growth of *Bifidobacteria* and *Lactobacilli*, whereas it can reduce the abundance of Enterobacteriaceae in a dose-dependent manner [45]. Pectin rich in rhamnogalacturonan-I which is refined from citrus segment membranes was demonstrated to significantly promote the production *Bifidobacteria*, *Lactobacilli*, and *Faecalibaculum* spp. in C57BL/6J male mice, and to be metabolized to SCFAs, which reduces the pH value in the intestine [46]. Another experiment with C57BL/6J female mice showed that administration of pectin extracted from orange peel relieved the development of *Citrobacter rodentium*-induced colitis, which may be because of the increase in the diversity of the gut microbiota [47]. Acacia gum, also known as gum Arabic, a branched-chain polysaccharide exudate gum mainly produced from *Acacia senegal*, is composed of arabinose and galactose [34]. Calame et al. found that the intake of acacia gum at 10 g/d was effective in increasing the abundance of *Bifidobacteria* and *Lactobacilli* in healthy human volunteers [48]. The results of an in vitro study by Rawi et al. showed that acacia gum significantly increased the abundance of *Bifidobacteria* while it inhibited that of *Clostridium histolyticum*, the bacterium which gives rise to gut dysbiosis. Moreover, acacia gum promoted the production of butyrate, which may also contribute to ameliorating the gut microbiota [49]. RSs, both insoluble and soluble, can be fermented and utilized by colonic microorganisms. They can increase the abundance of *Bacteroides*, *Bifidobacteria*, *Lactobacilli*, *Coprococcus*, and *Allobaculum* [50,51,52] and cooperatively perform their prebiotic effects with other active constituents such as chitosan oligosaccharide [70]. The effects of SDFs on the gut microbiota are summarized in Table 1.

Except for the different types and amounts of SDFs and other polysaccharides from food, dietary and endogenous proteins and mucins that come from intestinal epithelial cells are also nutrients for the gut microbiota [66,71]. The intestinal epithelium is covered and protected by a mucous layer to keep bacteria isolated from the mucosa [72]. SDFs and SCFAs stimulate mucus production and secretion [73]. Inadequate intake of SDFs will reduce the number of probiotics and transfer the metabolism of the gut microbiota to utilize amino acids in the other substrates, which can give rise to injury of the intestinal mucosa by accumulation of harmful metabolites, such as branched-chain fatty acids, ammonia, amines, N-nitroso compounds, and phenolic compounds [74]. Therefore, a dietary pattern with high sugar, fat, and protein but low fiber may result in the development of chronic inflammatory diseases such as IBD, CRC, allergies, cardiovascular disease, and obesity [75,76,77]. Sufficient intake of SDFs can protect the intestinal mucosa from degradation by intestinal bacteria and to a certain extent prevent these diseases.

Studying the impact of SDFs on health via the gut microbiota is sophisticated due to certain interferences. First, animal experiments often administer refined or modified SDFs in the form of feed additives. However, the physicochemical properties of SDFs such as viscosity or fermentability may be different when SDFs are eaten in the form of natural foods, thus altering their physiological functions. Second, natural foods rich in SDFs also contain other beneficial nutrients, such as minerals and phytochemicals, which make it difficult to determine the precise effects of SDFs alone on human health. Therefore, whether the results from animal experiments can be applied to humans requires further study.

## 5. SDFs and Their Metabolites Display Important Physiological Effects on Human Health

Although SDFs hardly directly provide energy for humans, SDFs per se exhibits specific physiological functions as recognized nutrients. In addition to stimulating the production and secretion of mucus, SDFs and SCFAs have other important physiological functions. In this section, we summarized the physiological functions of SDFs, including those that are attributed to SCFAs.

### 5.1. Increase Satiety and Reduce Energy Intake

Viscous SDFs retard the hydrolysis and absorption of energetic nutrients from food in the small intestine, such as starch and triglyceride. So, SDFs can significantly reduce the total intake of energy as well as glucose and cholesterol, so they contribute to slowing down the process of obesity, type 2 diabetes mellitus (T2DM), hyperlipidemia, and related metabolic diseases [78,79]. Then, the chyme gets to and stimulates the terminal ileum, where the mucosa responses and releases glucagon-like peptide-1 (GLP-1). The results from the experiments in human and pigs indicated that GLP-1 can inhibit gastric emptying and reduce intestinal peristalsis [80,81]. Therefore, viscous SDFs are helpful to control appetite, improve insulin sensitivity, and reduce weight.

### 5.2. Promote the Metabolism and Absorption of Active Substances

Many viscous SDFs can provide a platform for the metabolism of active substances. Take the bile acids as an example. Bile acids are produced in the liver and metabolized by enzymes from intestinal bacteria, which not only promote the absorption of dietary fat but also play indispensable roles in maintaining the healthy gut microbiota, balanced lipid and carbohydrate metabolism, insulin sensitivity, and innate immunity [82]. In the upper segment of the ileum and colon, conjugated primary bile acids combine with SDFs, where they are hydrolyzed to free primary bile acids by the bile salt hydrolase (BSH) from the intestinal bacteria, mainly *Bacteroides* and *Lactobacilli* [83]. Then, 7α-dehydroxylase also from the intestinal bacteria, such as *Clostridium* spp. and *Eubacterium* spp., catalyzes the free primary bile acids to secondary bile acids [84,85].

In addition to organic substrates, viscous SDFs can bind with inorganic nutrients such as metal ions. It was reported that after being bound with SDFs, calcium, magnesium, iron, copper, and zinc are transported to the distal colon. With the degradation of SDFs by the local bacteria, the ions are released and exhibit specific effects including resisting pathogens, increasing the diversity of the intestinal bacterial community, and protecting the gut from infection [76]. Furthermore, SCFAs produced via the gut microbiota fermenting SDFs combine with ions to form soluble salts, which are more prone to absorption by the colon [86,87].

### 5.3. SCFAs Act as Histone Deacetylase (HDAC) Inhibitors

Intracellular butyrate and propionate inhibit the activity of HDACs in colon cells and immune cells, leading to histone hyperacetylation, which in turn affects gene expression and cell differentiation, proliferation, and apoptosis [88]. Many studies have shown that SCFAs have important anti-inflammatory effects due to HDAC inhibition. For example, SCFAs can down-regulate proinflammatory cytokines such as interleukin-6 (IL-6) and IL-12 in colonic macrophages and differentiate dendritic cells from bone marrow stem cells [89,90]. Moreover, SCFAs can induce colonic regulatory T cells (Tregs) in mice [91]. There is also evidence that butyrate and propionate can induce the differentiation of Tregs, which can express the transcription factor Foxp3 via increasing the acetylation at the gene locus of foxP3. Foxp3 is found to play a crucial role in controlling intestinal inflammation in mice [92]. In addition, butyrate and propionate activate the AP-1 signaling pathway in human epithelial cells, which plays an important role in controlling proliferation and inducing apoptosis of colon cancer cells [93].

In brief, SCFAs, especially butyrate, not only provide the most energy for colon cells, but also aid to a large extent in the prevention of inflammation and CRC due to HDAC inhibition.

### 5.4. SCFAs Are Important Ligands for Specific G-Protein Coupled Receptors (GPCRs)

In addition to acting as HDAC inhibitors, SCFAs also exert important physiological functions as ligands for GPCRs. Three GPCRs (GPR41, GPR43, and GPR109A) involved in immune regulation were proven to specifically respond to free fatty acids. Therefore, GPR43 and GPR41 were also named FFAR (free fatty acid receptor) 2 and FFAR3, respectively [94].

In mice, butyrate can increase the secretion of Tregs, IL-18, and T cells producing IL-10 in intestinal epithelial cells via stimulating GPR109A [95]. Additionally, the study by Macia et al. in mice indicated that SCFAs derived from a high-fiber diet stimulated GPR43 and GPR109A to activate the NLRP3 inflammasome, which produces IL-18. This effect maintains intestinal homeostasis by decreasing the inflammatory response of the gut and maintaining the integrity of the mucosal barrier, which prevents bacterial invasion and infection [96]. In the intestine and white adipose tissue (WAT) of mice, SCFA-dependent GPR43 stimulation (especially acetate and propionate) displays beneficial effects in ameliorating the metabolism of glucose and lipids by GLP-1 secretion and anti-lipolytic activity, respectively [97]. GPR41 also plays a role in the regulating appetite. Samuel et al. reported that by binding with GPR41, SCFAs induce the production of peptide YY, which inhibits gastrointestinal motility and gastric acid secretion in mice [98].

The above studies indicated that SCFAs have significant immunologic and metabolic functions as ligands for GPCRs, affecting the incidence of IBD, CRC, and other cancers as well as chronic metabolic diseases.

**Figure 3:**
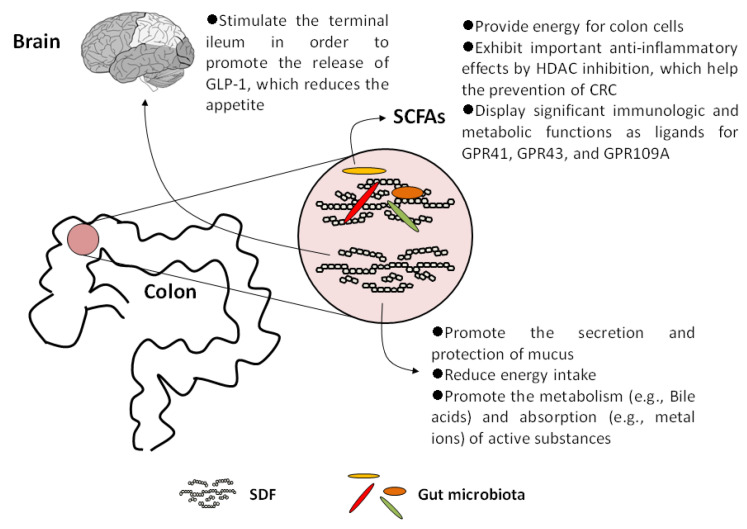
Summary of identified physiological effects of SDF and SCFAs on human health.

The identified physiological effects of SDFs and SCFAs on human health are summarized in Figure 3.

## 6. Safety of SDFs

Although SDFs show excellent health effects, it cannot be ignored that inappropriate SDF intake may lead to certain health hazards, which depend on the type and quantity of SDFs as well as the physiological background of the host.

First, a sudden increase of SDF intake, even when consumed judiciously, may lead to abdominal distension, flatulence, constipation, diarrhea, and other syndromes of IBS [99,100]. Second, as mentioned above, the binding by SDFs can promote to a certain extent the absorption of certain micronutrients such as some metal ions in the colon. However, the study on six healthy young women by Riedl et al. indicated that the bioavailability of β-carotene, lycopene, and lutein, was markedly reduced by three different kinds of SDFs, pectin, guar gum, and alginate [101]. This suggests that excessive SDF intake may be disadvantageous to certain people with micronutrient deficiencies. In addition, Bruggencate et al. reported that the rapid fermentation of FOS by endogenous microbiota damaged the intestinal mucosal barrier and increased intestinal permeability, which caused pathogen infection in rats [102]. Moreover, the study by Singh et al. found that formulating a diet rich in refined inulin or other SDFs to feed TLR5-deficient (*T5KO*) mice with obesity caused by dysregulated gut microbiota, about 40% of mice had a lower weight than before. However, many mice suffered from icteric hepatocellular carcinoma (HCC). Further research revealed that SDF-induced HCC in mice developed via elevation of secondary bile acids in the systemic circulation, cholestasis, and hepatocyte death, followed by neutrophilic inflammation of the liver. Furthermore, fecal microbiota analysis by 16S rRNA sequencing showed that the HCC-prone mice exhibited gut dysbiosis characterized by a loss in species richness and diversity and an increase in the Proteobacteria phylum and the fiber-utilizing microbes including *Clostridium* spp. However, such HCC in T5KO mice cannot be induced by cellulose, the insoluble and non-fermentable fiber, and it was not observed in germ-free nor antibiotics-treated mice [103]. Although acting as a kind of metabolite with important physiological functions, certain bile acids, especially secondary bile acids, were proven to be cytotoxic and cancer-promoting, which have adverse effects on the structure and function of the colonic epithelium by many mechanisms including DNA oxidative damage, inflammation, NF-κB activation, reducing apoptosis, and differentiation, as well as enhancing cell proliferation [104]. Apart from HCC, the changed bile acid profile derived from dysregulated gut microbiota was demonstrated to be associated with a variety of digestive diseases. For example, many patients suffering from CRC exhibited abnormal bile acid metabolism characteristic of redundant secondary bile acids. These redundant secondary bile acids, including deoxycholic acid, lithocholic acid, and taurochenodeoxycholic acid, originate in the aberrant elevation of certain gut bacteria expressing BSH and 7α-dehydroxylase, whose proportion is significantly higher than that in the gut microbiota of healthy people [82]. These facts hinted that, despite notable physiological benefits, fortification of diets with SDFs should be done with great caution as it may cause severe digestive disorders, especially under the background of dysregulated gut microbiota.

## 7. Conclusions and Prospects

Studies have confirmed that the gut microbiota is closely related to human health. The results from experiments with mice showed that the genetic background contributed to less than 12% of the difference of the gut microbiota, but dietary structure and habits contributed to 57% [105]. SDFs have a significant advantage in improving the gut microbiota due to their high fermentation efficiency. Furthermore, many prospective cohort studies showed that the diversity of the gut microbiota is negatively correlated with the incidence rate of many chronic diseases, including IBD, CRC, obesity, and T2DM [106,107,108,109,110]. In view of the fact that specific SDFs can promote the proliferation of specific intestinal bacteria, a diet rich in various SDFs is beneficial to health. A reasonable combination of various SDFs by raw food is undoubtedly helpful to improve the gut microbiota. In addition, studies also showed that the host response to SDF intervention is personalized, and the results to a significant extent depend on the individual’s intestinal ecology before treatment [15]. Therefore, based on the precise analysis of host gut ecology, the personalized treatment of dietary intervention with SDFs combined with antibiotic therapy and/or fecal microbial transplantation (FMT), may be effective in improving health, especially in the prevention and treatment of intestinal diseases. SDFs have a promising future by increasing the community of beneficial microorganisms and their products of growth and metabolism in the host. Notably, certain prospective cohort studies showed that insoluble fibers in diet is more protective against some metabolic diseases (e.g., T2DM) than SDFs [111]. At the same time, considering the side effects of SDFs via dysregulated microbial fermentation, precise SDF intake as well as a reasonable association with insoluble dietary fiber on the basis of the background of the gut microbiota in hosts is of great importance in achieving the goal of disease prevention and cure. With the development of microbiome analysis, the functions of intestine ecology will be clearer. The precise understanding of metabolic pathways and end products involved with the utilization of SDFs and other nutrients by the gut microbiota is the hotspot of the present research. There is an urgent need to elucidate the interactions between bacterial strain levels and specific types of SDFs, which may undoubtedly help improve human health by accurately determining the diversity and functions of the gut microbiota.

## Data Availability

Not applicable.
